# Supplementary material for: Acquired and intrinsic resistance to vemurafenib in BRAFV600E ‐driven melanoma brain metastases
Source: FEBS Open Bio. 2023 Nov 30;14(1):96–111. doi: 10.1002/2211-5463.13730 (PMC10761933; doi:10.1002/2211-5463.13730)
Supplement: Supplementary file 1 — Fig. S1. Body weight curves from intervention studies presented in Fig. 2. (A) Average mouse body weights from the experiment presented in Fig. 2A‐B. Treatment periods are shaded in gray. Data are represented as mean ± SD (n ≥ 7). (B) Average mouse body weights from the experiment presented in Fig. 2D. Treatment period is shaded in gray. Data are represented as mean ± SD (n ≥ 7). (C) Average mouse body weights from the experiment presented in Fig. 2E‐F. Treatment period is shaded in gray. Data are represented as mean ± SD (n ≥ 8). [file FEB4-14-96-s001.pdf]

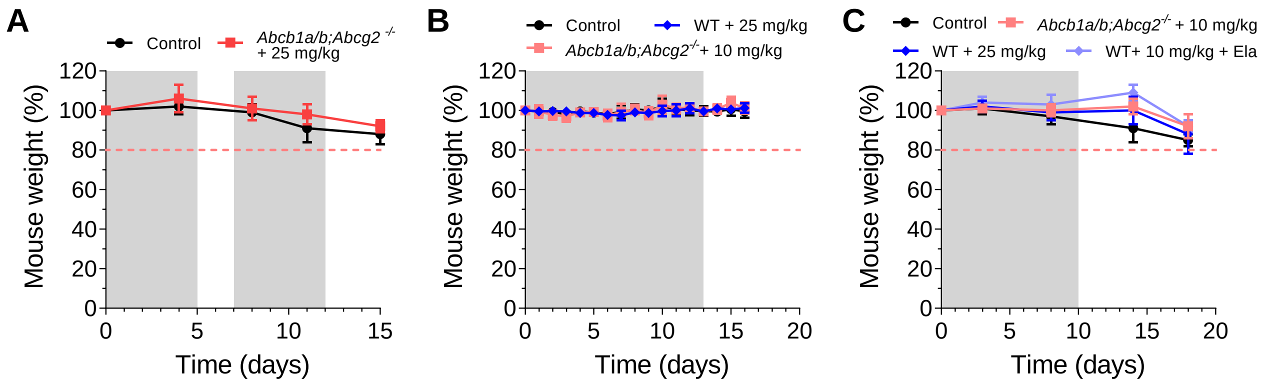

Supplementary Figure 1. Body weight curves from intervention studies presented in Figure 2. (A) Average mouse body weights from the experiment presented in Figure 2A-B. Treatment periods are shaded in grey. Data are represented as mean ± SD (n ≥ 7). (**B**) Average mouse body weights from the experiment presented in Figure 2D. Treatment period is shaded in grey. Data are represented as mean ± SD (n ≥ 7). (**C**) Average mouse body weights from the experiment presented in Figure 2E-F. Treatment period is shaded in grey. Data are represented as mean ± SD (n ≥ 8).
